# TRIM family proteins: dual roles in tumor immunity

**DOI:** 10.3389/fcell.2026.1818014

**Published:** 2026-05-13

**Authors:** Runjie Cai, Liang Chen

**Affiliations:** 1 Department of Biomedical Engineering, Southern University of Science and Technology, Shenzhen, Guangdong, China; 2 Shenzhen Laboratory of Tumor Cell Biology, Center for Protein and Cell-based Drugs, Institute of Biomedicine and Biotechnology, Shenzhen Institute of Advanced Technology, Chinese Academy of Sciences, Shenzhen, China; 3 Molecular Cancer Research Center, School of Medicine, Shenzhen Campus of Sun Yat-Sen University, Sun Yat-Sen University, Shenzhen, China

**Keywords:** cGAS-STING, immune checkpoint, TRIM family proteins, tumor immunology, tumor microenvironment, TME, ubiquitination

## Abstract

Tripartite motif (TRIM) proteins represent one of the largest subfamilies of E3 ubiquitin ligases and exert complex yet critical dual roles in tumor immunology by orchestrating protein ubiquitination. This review systematically delineates the multifaceted regulatory roles of TRIM proteins in tumor immunity, with a focus on their dual functions within the tumor immune microenvironment, particularly regarding immune checkpoints and tumor-infiltrating immune cells. Notably, the same TRIM protein may exert opposing immunomodulatory effects in different tumor types or microenvironmental contexts, underscoring the critical influence of context on their functions. This article aims to synthesize current advances in understanding the dual roles of the TRIM family in tumor immunity and to discuss their potential and challenges as novel targets for immunotherapy.

## 1 Introduction

Cancer presents a formidable global public health challenge, with its burden growing increasingly severe. Global cancer cases are projected to reach 28.4 million by 2040, a 47% increase from 2020 (Bray et al., 2018). In response to this growing threat, cancer therapeutics has evolved from conventional chemotherapy and radiotherapy into a diversified and personalized paradigm. Targeted therapies or immunotherapies such as kinase inhibitors targeting specific mutant proteins, monoclonal antibody-based drugs, and chimeric antigen receptor T-cell (CAR-T) therapies have significantly improved clinical outcomes for some patients (Gotwals et al., 2017; Stine et al., 2021; Liu B. et al., 2024). Nevertheless, current treatment modalities often fail to achieve complete tumor eradication, largely due to the intricate molecular heterogeneity and complex immune regulatory networks underlying tumorigenesis and disease progression (Wang et al., 2018; Mullard, 2020). In this context, protein post-translational modifications (PTMs), which function as precise molecular switches regulating protein activity, stability, and interactions, are increasingly recognized as central players in almost all hallmarks of cancer (Mani et al., 2022). Among these PTMs, ubiquitination orchestrates the fate of target proteins through a defined enzymatic cascade involving ubiquitin-activating (E1), ubiquitin-conjugating (E2), and ubiquitin ligase (E3) enzymes, with E3 ubiquitin ligases playing a pivotal role by conferring substrate specificity (Zhao et al., 2021). Consequently, targeting specific E3 ubiquitin ligases within tumor cells or immune cells has emerged as a promising therapeutic strategy to intervene in dysregulated ubiquitination and remodel anti-tumor immunity.

The tripartite motif (TRIM) family constitutes one of the largest and most functionally significant subclasses of E3 ubiquitin ligases. Comprising over 80 members encoded in the human genome, TRIM proteins are defined by a conserved N-terminal tripartite motif consisting of a RING finger domain, one or two B-box domains (B1 and/or B2), and a coiled-coil (CC) domain (Ozato et al., 2008; Gushchina et al., 2018). The RING finger domain contains a Cys3HisCys4 amino acid motif that coordinates two zinc atoms and plays a crucial role in the ubiquitination pathway, such as recruiting ubiquitin-conjugating enzymes (E2s) (Hatakeyama, 2011; Li et al., 2014). The B-box domain contains one or two zinc-binding motifs, typically designated B1 and B2, although some TRIM members possess only a B2 domain (Hatakeyama, 2011; Li et al., 2014). The coiled-coil domain, well-conserved across all TRIM proteins and usually positioned after B-box2, is primarily involved in homo-oligomerization, formation of macromolecular complexes, and subcellular localization. Additionally, the highly variable C-terminal domains (e.g., PRY-SPRY, BROMO, NHL) confer unique substrate recognition capabilities and functional specificity to individual TRIM proteins (Reymond et al., 2001; Zhan and Zhang, 2021).

TRIM proteins regulate a broad spectrum of fundamental cellular processes, including cell proliferation, apoptosis, autophagy, innate immune responses, and DNA damage repair (Eames et al., 2012; Versteeg et al., 2014; Hatakeyama, 2017; Shen et al., 2019; Xu et al., 2019). Our group has demonstrated that several members of the TRIM family, including TRIM11, TRIM25 and TRIM28, play significant roles in promoting anti-tumor immunity and regulating cellular protein homeostasis (Chen et al., 2017; Chen et al., 2018; Liu et al., 2020; Zhang et al., 2020; Liu et al., 2021; Zhang et al., 2022; Xu et al., 2025). For example, TRIM11 promotes antitumor immunity in the tumor microenvironment by facilitating JAK1 ubiquitination through its E3 ligase activity, thereby preventing IFN-γ-induced PD-L1 upregulation (Xu et al., 2025). This finding contrasts with the previously reported oncogenic role of TRIM11 in various cancers, underscoring its context-dependent dual functions in tumorigenesis. Accumulating evidence indicates that many TRIM proteins are dysregulated in diverse solid tumors and hematological malignancies. Through their ubiquitination functions, these proteins participate directly or indirectly in multiple tumor-immunity regulatory pathways, exerting either pro-tumor or anti-tumor effects (Hatakeyama, 2011; Mohammadi et al., 2022). In this review, we focus on the dual regulatory roles of the tripartite motif (TRIM) family in tumor immunology. The functional landscape of the TRIM family is defined by three fundamental dimensions: cell-type context, substrate specificity, and the signaling microenvironment. First, lineage-dependent effects often result in antithetical outcomes. For instance, TRIM21 facilitates PD-L1 degradation in tumor cells to enhance immunosurveillance, yet stabilizes surface PD-1 in T cells to promote exhaustion. Second, substrate specificity allows individual TRIM proteins to regulate divergent pathways within the same cell type; for example, TRIM29 modulates PD-L1 stability in certain malignancies but targets TAB2 to suppress NK cell cytotoxicity. Finally, the signaling context, such as the presence of inflammatory cytokines, can fundamentally reprogram TRIM functions by inducing shifts in their subcellular localization or protein-protein interactions. By integrating these variables, this review establishes a systematic framework to resolve the complex regulatory networks through which the TRIM family modulates the balance between immune activation and escape.

A deeper understanding of the precise functions and mechanisms of TRIM proteins in specific tumor contexts will not only help clarify the importance and complexity of the TRIM family in tumor-immunity networks, but also provide a critical theoretical foundation and novel molecular targets for developing next-generation targeted immunotherapies—particularly emerging protein-targeting technologies such as proteolysis-targeting chimeras (PROTACs).

## 2 TRIMs involved in enhancing anti-tumor immunity

### 2.1 Effects on the tumor microenvironment (TME)

**FIGURE 1:**
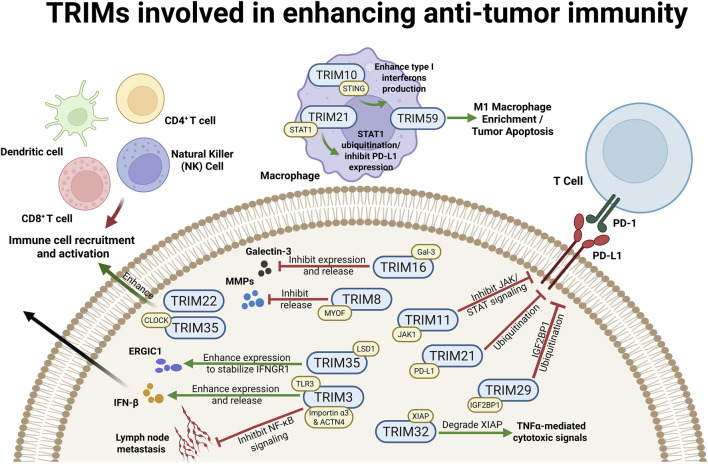
TRIM proteins involved in enhancing anti-tumor immunity. The schematic illustrates TRIM-mediated mechanisms enhancing anti-tumor immunity. Green and yellow modules denote complex formation and ubiquitination substrates, respectively; unannotated proteins indicate unvalidated targets. Left and Center (TME Remodeling and Cytokine Signaling): In the TME, TRIM3 ubiquitinates TLR3 to enhance IFN-β release, and targets Importin α3/ACTN4 to inhibit NF-κB-mediated metastasis. Immune cell recruitment is promoted by TRIM22 and TRIM35, which ubiquitinates CLOCK and LSD1 (stabilizing ERGIC1/IFNGR1). In macrophages, TRIM10 and TRIM21 ubiquitinate STING and STAT1 to enhance IFN-I production and inhibit PD-L1 expression, respectively. TRIM59 promotes M1 macrophage enrichment and tumor apoptosis *via* unconfirmed pathways. Tumor progression is further impeded by TRIM8 (ubiquitinating MYOF to inhibit MMPs), TRIM16 (ubiquitinating Gal-3 to prevent T cell cuproptosis), and TRIM32 (ubiquitinating XIAP to enhance TNFα cytotoxicity). Right (Immune Checkpoint Regulation): the PD-L1 immunosuppressive axis is dismantled *via* ubiquitination by TRIM11 (targeting JAK1 to inhibit STAT signaling), TRIM21 (targeting PD-L1 directly for degradation), and TRIM29 (targeting IGF2BP1 to destabilize PD-L1 transcripts). Created in BioRender. Cai, R. (2026) https://BioRender.com/8tkluoc.

The tumor microenvironment (TME) constitutes a physicochemical and biological niche co-opted by neoplastic cells to support proliferation and evade immunosurveillance. Emerging evidence highlights the tripartite motif (TRIM) protein family as a critical regulator of antitumor immunity through TME remodeling (Figure 1).

#### 2.1.1 Promoting the recruitment and survival of effector lymphocytes

Several TRIM family members actively reconfigure the TME to promote the recruitment and persistence of effector lymphocytes. In glioma models, upregulated TRIM16 mediates the degradation of extracellular Galectin-3 (Gal-3). This targeted clearance prevents Gal-3-induced aberrant copper uptake, effectively shielding CD8^+^ T lymphocytes from cuproptosis and preserving their survival and cytotoxic function (Wang et al., 2024). TRIM35 exhibits context-dependent immune modulation by targeting distinct substrates. In NSCLC, it catalyzes the K63-linked non-proteolytic ubiquitination of LSD1, preserving activating histone marks that stabilize membrane IFNGR1 to drive the recruitment of CD8^+^ cytotoxic T lymphocytes (Tang et al., 2023). Concurrently, in DLBCL, TRIM35 ubiquitinates the circadian regulator CLOCK, alleviating its suppression of the microenvironment and enhancing natural killer (NK) cell infiltration. Beyond recruitment, protecting infiltrating immune cells from metabolic toxicity is vital for sustained surveillance (Tan et al., 2021).

#### 2.1.2 Activating inflammatory pathways and restricting tumor stroma

TRIM proteins also intercept tumor-extrinsic signaling to establish an immunostimulatory stroma. Exosomal TRIM3 functions as a pivotal tumor suppressor by orchestrating communication between malignant cells and innate immune sentinels (Fu et al., 2018). By interacting with Toll-like receptor 3 (TLR3) and promoting its K63-linked polyubiquitination, TRIM3 sustains IFN-β production, reprogramming the TME toward a state enriched with CD4^+^ T cells, M1 macrophages, and NK cells (Xu et al., 2026). Additionally, in gastric and esophageal squamous cell carcinomas, TRIM3 targets Importin α3 and α-Actinin-4 for proteasomal degradation (Zhu et al., 2019). This event disrupts the constitutive activation of NF-κB, which consequently suppresses the upregulation of lymphangiogenic factors and restricts peritumoral lymphatic expansion. This pro-inflammatory network is further reinforced by TRIM8, which operates downstream of macrophage activation. Induced by M1 macrophages, TRIM8 functions as an effector of metastasis suppression by targeting the membrane repair protein Myoferlin (MYOF) for K48-linked polyubiquitination and degradation (Wei et al., 2025). The subsequent abrogation of MYOF restricts the secretion of matrix metalloproteinases, attenuating the invasive capacity of lung cancer cells and highlighting a cooperative defense axis between innate immunity and TRIM ligase activity.

### 2.2 Effects on immune checkpoints

Immune checkpoints are inhibitory signaling pathways that modulate immune response intensity, prevent tissue damage, and maintain self-tolerance (He and Xu, 2020). Immune checkpoint blockade (ICB) has demonstrated considerable clinical benefit in monotherapy and combination regimens. Recent research on TRIM proteins in tumor immunotherapy has increasingly focused on their mechanisms of action against immune checkpoints, particularly PD-L1 (Figure 1).

#### 2.2.1 Mediating the proteasomal degradation of PD-L1

TRIM21 serves as a central E3 ligase governing the direct proteasomal turnover of PD-L1 across multiple malignancy models. In lung cancer environments, the physical interaction between TRIM21 and PD-L1 is scaffolded by the long non-coding RNA LINC02418. The stability of this molecular scaffold is regulated by the METTL3/YTHDF2 m^6^A modification axis; consequently, the depletion of LINC02418 impairs TRIM21-mediated PD-L1 degradation and fosters immune escape (Sun et al., 2023). Furthermore, in lung adenocarcinoma, the interaction between TRIM21 and its substrate is sensitive to oncogenic signaling, as Cyclin-dependent kinase 5 (CDK5) can physically impede this binding (Gao et al., 2021). Inhibition of CDK5 restores TRIM21 activity, promoting robust PD-L1 degradation and reinforcing anti-tumor immunity.

#### 2.2.2 Transcriptional and post-transcriptional suppression of PD-L1

Beyond direct proteolysis, specific TRIM proteins dismantle immune checkpoints by targeting upstream signaling kinases or essential mRNA stabilizers. TRIM11 operates as a molecular switch for PD-L1 expression by modulating the IFN-γ/JAK/STAT axis (Xu et al., 2025). By catalyzing the K63-linked polyubiquitination of JAK1, TRIM11 inhibits its kinase function and downstream STAT signaling, effectively blunting IFN-γ-induced PD-L1 transcription. Parallelly, in gastric cancer environments, TRIM29 modulates checkpoint levels by targeting the RNA-binding protein IGF2BP1 (Jiang T. et al., 2024). Given that IGF2BP1 normally protects PD-L1 mRNA from m^6^A-dependent decay, its TRIM29-mediated ubiquitination leads to the destabilization of PD-L1 transcripts. This post-transcriptional intervention relieves T cell suppression and correlates with a more favorable immune landscape in clinical cohorts.

### 2.3 Effects on immune cells

Beyond modulating checkpoint expression, TRIM proteins function as intrinsic determinants of immune cell signaling, directly shaping the phenotypic landscape and effector potential of tumor-infiltrating populations (Figure 1).

#### 2.3.1 Modulating macrophage polarization and signaling pathways

TRIM family members are essential for maintaining the functional equilibrium of tumor-associated macrophages (TAMs). In gastric cancer environments, TRIM21 promotes immune homeostasis by targeting STAT1 for proteasomal degradation (Zhang et al., 2025). This regulatory axis prevents the excessive accumulation of STAT1 and the subsequent upregulation of CCL8 and PD-L1, which would otherwise induce CD8^+^ T cell exhaustion. However, high expression of the receptor TREM2 can competitively disrupt the TRIM21–STAT1 interaction, highlighting the vulnerability of this anti-tumor mechanism to microenvironmental subversion. Similarly, within melanoma microenvironments, TRIM59 functions as a metastatic surveillance factor in M2 macrophages by suppressing PI3K and ERK signaling. The downregulation of TRIM59 relieves this enzymatic inhibition, promoting the secretion of matrix metalloproteinases (MMPs) and Madcam-1, which collectively remodel the extracellular matrix to favor tumor dissemination (Tian et al., 2019a).

#### 2.3.2 Sensitizing tumor cells to immune-mediated cytotoxicity

Specific TRIM proteins confer unique effector functions upon immune cells to facilitate direct tumor elimination. Following *Bacillus* Calmette-Guérin (BCG) stimulation in fibrosarcoma models, TRIM59 is upregulated on the surface of activated macrophages (Tian et al., 2019b). In this specific context, TRIM59 mediates direct pro-apoptotic effects on tumor cells through physical contact rather than the secretion of soluble mediators. This identifies a novel effector role for the TRIM family in innate immunity, where these proteins act as surface-bound executors of tumor suppression, independent of canonical cytokine-mediated pathways. This multi-dimensional immunomodulatory role further extends to lymphocyte populations. In colon adenocarcinoma, TRIM22 expression has been identified as a critical determinant of improved prognosis; it significantly promotes the infiltration of lymphocytes and dendritic cells within the tumor tissue by participating in cytokine receptor interactions and lymphocyte activation processes (Guo et al., 2021). Furthermore, TRIM32 sensitizes tumor cells to immune-mediated killing by ubiquitinating and degrading the anti-apoptotic protein XIAP, thereby overcoming resistance to TNFα-induced cytotoxicity and facilitating more efficient immune clearance (Ryu et al., 2011).

## 3 TRIMs involved in promoting tumor immune escape

### 3.1 Effects on the tumor microenvironment (TME)

**FIGURE 2:**
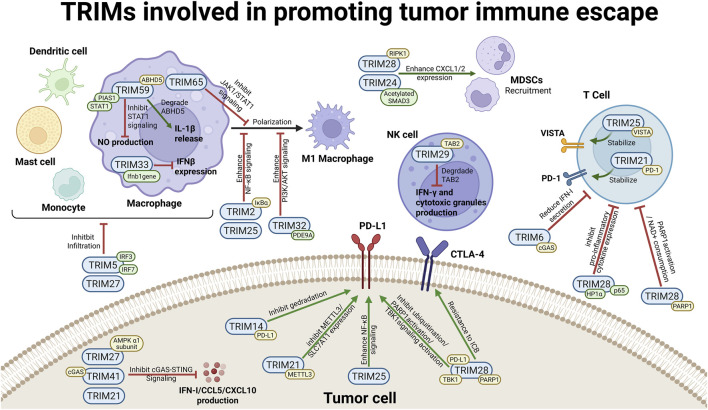
TRIM proteins involved in promoting tumor immune escape. The schematic illustrates TRIM-mediated mechanisms facilitating immune evasion. Green and yellow modules denote complex formation and ubiquitination substrates, respectively; unannotated proteins indicate unvalidated or bioinformatics-derived targets. Left (Immune Cells Modulation): In macrophages, TRIM59 complexes with PIAS1 to inhibit STAT1 and ubiquitinates ABHD5 to trigger IL-1β release. TRIM33 directly binds the Ifnb1 gene, while TRIM65 inhibits JAK1/STAT1 signaling. Immunosuppressive M2 polarization is driven by TRIM2 (ubiquitinating IκBα) and TRIM32 (complexing with PDE9A). TRIM5 (complexing with IRF3/IRF7) and TRIM27 inhibit monocyte infiltration. Top (MDSC and NK Cell Regulation): To recruit MDSCs, TRIM24 (complexing with acetylated SMAD3) and TRIM28 (ubiquitinating RIPK1) enhance CXCL1/2 expression. In NK cells, TRIM29 ubiquitinates TAB2 to suppress cytotoxicity. Right (T Cell Suppression): On the T cell surface, TRIM25 and TRIM21 stabilize VISTA and PD-1 *via* ubiquitination. Intracellularly, the cGAS-STING pathway is inhibited by TRIM6 and TRIM41 (ubiquitinating cGAS) and TRIM27 (ubiquitinating AMPK α1), reducing IFN-I and chemokine production. Finally, tumor PD-L1 levels are upregulated to promote ICB resistance *via* TRIM14 (ubiquitinating PD-L1), TRIM21 (ubiquitinating METTL3), TRIM28 (ubiquitinating PARP1/TBK1 and complexing with HP1α/p65), and TRIM25. Created in BioRender. Cai, R. (2026) https://BioRender.com/cbdc0xk.

In the context of tumor immune escape, the tripartite motif (TRIM) family serves as a molecular architect of the immunosuppressive niche, primarily by driving the functional reprogramming of myeloid cells and orchestrating the influx of suppressive populations (Figure 2).

#### 3.1.1 Inducing M2 macrophage polarization and immunosuppression

The induction of an M2-like, pro-tumorigenic macrophage phenotype is a hallmark of TRIM-mediated TME remodeling. In colorectal and gastric cancer environments, TRIM2 and TRIM32 function as pivotal rheostats for macrophage plasticity. Epinephrine-upregulated TRIM2 acts as an E3 ligase to degrade IκBα, triggering sustained NF-κB activation that polarizes tumor-associated macrophages (TAMs) toward an M2 phenotype (Liu Z. et al., 2024). Similarly, TRIM32 interacts with PDE9A to activate the PI3K/AKT signaling axis, which recruits and polarizes TAMs to attenuate CD8^+^ T cell effector functions (Wang C. et al., 2025). This myeloid-mediated suppression is further reinforced in glioma models by TRIM25, which utilizes the NF-κB axis to couple apoptosis resistance with macrophage-driven immunosuppression (Ge et al., 2022). Beyond direct signaling, the absence of RIPK3 in various solid tumors allows TRIM28 to repress immunostimulatory genes, a process that maintains an inhibitory milieu by preventing the induction of pro-inflammatory cytokines like TNF and IFN-β (Park et al., 2021).

#### 3.1.2 Promoting MDSC recruitment via chemokine signaling

The recruitment of myeloid-derived suppressor cells (MDSCs) constitutes a primary barrier to immunosurveillance, often mediated by TRIM-dependent chemokine signaling. In triple-negative breast cancer models, the TRIM24–SMAD3 transcriptional complex—scaffolded by KAT6A-mediated SMAD3 acetylation—enhances the expression of CXCL2 to facilitate MDSC influx and metastasis (Yu et al., 2021). Parallelly, in non-small cell lung cancer (NSCLC) environments, TRIM28 interacts with RIPK1 to catalyze K63-linked ubiquitination, activating NF-κB-dependent CXCL1 upregulation. The resulting CXCL1/CXCR2 signaling axis drives robust MDSC recruitment, which not only impairs CD8^+^ T cell infiltration but also promotes resistance to anti-PD-1 therapy (Liang et al., 2023). Furthermore, the strategic reconfiguration of the TME involves the modulation of broader immune cell profiles, as evidenced by the correlation between TRIM5 (Chen et al., 2020) and TRIM27 (Liang et al., 2025) expression and the altered infiltration of monocytes and natural killer cells in glioma and melanoma, respectively.

### 3.2 Effects on immune checkpoints

To subvert host immunosurveillance, malignancies frequently hijack specific tripartite motif (TRIM) proteins to reinforce immune checkpoint signaling. These E3 ligases predominantly sustain programmed death-ligand 1 (PD-L1) expression through complex post-translational modifications or the activation of upstream transcriptional networks (Figure 2).

#### 3.2.1 Stabilizing PD-L1 via post-translational modifications

TRIM proteins exploit non-degradative ubiquitin linkages and crosstalk with other post-translational modifications to protect PD-L1 from intracellular clearance. In breast cancer models exhibiting resistance to IFNα therapy, TRIM14 acts as an essential stabilizer of PD-L1 (Liu D. et al., 2024). Rather than directly ubiquitinating the checkpoint, TRIM14 recruits the deubiquitinase USP14 to strip K63-linked ubiquitin chains from PD-L1. This targeted deubiquitination prevents p62/SQSTM1-mediated autophagic degradation, enforcing PD-L1 accumulation and subsequent CD8^+^ T cell suppression. Concurrently, in gastric cancer environments, TRIM28 utilizes a direct physical interaction *via* its B-box2 domain to suppress the ubiquitination of PD-L1 while enhancing its SUMOylation (Ma et al., 2023). This dual regulatory capacity establishes a highly stable checkpoint barrier on the tumor surface.

#### 3.2.2 Driving PD-L1 transcription and adaptive immune resistance

Beyond stabilizing existing checkpoint proteins, TRIM members orchestrate the transcriptional upregulation of PD-L1 to drive adaptive immune resistance. TRIM28 exhibits profound mechanistic pleiotropy in this context. It catalyzes the K63-linked ubiquitination of TBK1, diverting the signal to activate TBK1–IRF1 and TBK1–mTOR pathways, which robustly increase PD-L1 transcription (Ma et al., 2023). In clear cell renal cell carcinoma, TRIM28 facilitates the SUMOylation of PARP1. This stabilization hyperactivates the NAD^+^–SIRT1 pathway to enhance PD-L1 expression, while the parallel depletion of nuclear NAD^+^ intrinsically compromises CD8^+^ T cell function (Zhan et al., 2025). Other TRIM members couple checkpoint expression to cellular stress and survival pathways. In glioma models, TRIM25 functions as an oncogenic hub that utilizes the NF-κB signaling axis to concurrently inhibit apoptosis and upregulate PD-L1, consolidating macrophage-mediated immunosuppression (Ge et al., 2022). Furthermore, TRIM21 reveals a paradigm of adaptive resistance in pancreatic ductal adenocarcinoma (PDAC) environments (Mao et al., 2025). By targeting the m^6^A methyltransferase METTL3 for K48-linked degradation, TRIM21 destabilizes SLC7A11 mRNA and induces profound ferroptotic stress. This metabolic catastrophe remodels the tumor microenvironment, resulting in a compensatory elevation of PD-L1 levels that paradoxically sensitizes these tumors to anti-PD-1 immunotherapy.

### 3.3 Effects on immune cells

Beyond restructuring the extracellular milieu, specific tripartite motif (TRIM) proteins intrinsically paralyze immune cell function, acting as intracellular checkpoints that enforce immune evasion (Figure 2).

#### 3.3.1 Stabilizing inhibitory receptors and suppressing lymphocyte cytotoxicity

To subvert adaptive and innate cytotoxicity, TRIM proteins stabilize inhibitory surface receptors or dismantle essential activation cascades within lymphocytes. TRIM21 negatively regulates T cell immunity by catalyzing the K63-linked polyubiquitination of PD-1. This non-degradative modification antagonizes PD-1 turnover, sustaining high surface expression levels and persistently suppressing CD8^+^ T cell cytotoxicity (Shi et al., 2025). Similarly, TRIM25 stabilizes the inhibitory checkpoint VISTA on T lymphocytes *via* K63-linked ubiquitination, a molecular event enhanced by ERK-mediated phosphorylation, effectively establishing a robust immunosuppressive barrier (Sun et al., 2025). In natural killer (NK) cells, TRIM29 functions as an inducible negative feedback regulator that restrains innate immunosurveillance. Following stimulation with IL-12 and IL-18, upregulated TRIM29 targets the TAK1-binding protein 2 (TAB2) for K48-linked polyubiquitination and subsequent proteasomal degradation. The targeted destruction of TAB2 dampens downstream NF-κB and MAPK signaling cascades, severely limiting IFN-γ production and cytolytic activity (Dou et al., 2019). Conversely, the specific genetic ablation of TRIM29 unleashes NK cell effector functions, effectively restricting tumor growth and metastasis in melanoma models.

#### 3.3.2 Repressing anti-tumor inflammatory signaling in myeloid cells

Within myeloid populations, TRIM proteins subvert intrinsic signaling to abrogate anti-tumor inflammation and promote therapeutic resistance. TRIM33 functions as a critical epigenetic repressor that limits the efficacy of radiotherapy. Acting as a chromatin reader, TRIM33 directly represses the transcription of Ifnb1, maintaining a locally suppressed immune environment and preventing the establishment of CD8^+^ T cell-mediated immunological memory following radiation (Assouvie et al., 2025). TRIM59 demonstrates profound mechanistic versatility by modulating macrophage phenotypes through divergent intra- and intercellular pathways. Intracellularly, inflammatory stimuli drive the nuclear translocation of TRIM59, culminating in the formation of a TRIM59–PIAS1–STAT1 complex (Su et al., 2020). This physical interaction potentiates PIAS1-mediated inhibition of STAT1, completely abrogating the production of essential anti-tumor effectors such as nitric oxide and TNFα. Intercellularly, lung cancer-derived exosomes deliver TRIM59 directly into adjacent macrophages, where it degrades ABHD5, a recognized negative regulator of the NLRP3 inflammasome (Liang et al., 2020). The resulting aberrant inflammasome activation and IL-1β release reciprocally enhance tumor proliferation and invasiveness. Furthermore, in hepatocellular carcinoma environments, high expression of TRIM65 in tumor-associated macrophages inhibits the JAK1/STAT1 signaling axis, actively preventing immunostimulatory M1 polarization and compromising overall immune surveillance (Jiang M. et al., 2024).

## 4 TRIM-mediated regulation of the cGAS-STING innate sensing axis

A cornerstone of the TRIM-mediated immunomodulatory network is the regulation of innate sensing pathways, most notably the cGAS-STING axis. As the primordial surveillance system linking the detection of cytosolic DNA to potent adaptive anti-tumor responses, the cGAS-STING pathway serves as a decisive regulatory hub that determines the fundamental “inflammatory” state of the TME (Wang Q. et al., 2025). Recent investigations reveal that the TRIM family employs a sophisticated tactical repertoire to modulate this axis, ranging from the direct proteolysis of DNA sensors to the epigenetic silencing of downstream interferons. By intervening at multiple nodes, these E3 ligases ensure comprehensive control over innate sensing, offering a promising pharmacological frontier to restore the endogenous adjuvanticity of the tumor microenvironment (Figures 1, 2).

### 4.1 Promoting innate sensing via STING activation

While many TRIM proteins are characterized as suppressors of innate immunity, specific members are indispensable for signal transduction. TRIM10 functions as a critical positive regulator by orchestrating the spatial organization of the STING signalosome (Kong et al., 2023). Upon DNA sensing, TRIM10 facilitates the aggregation of STING within the Golgi apparatus, a localization step essential for its subsequent recruitment of TANK-binding kinase 1 (TBK1) and the phosphorylation of interferon regulatory factor 3 (IRF3). Furthermore, TRIM5 has been implicated in glioma as an orchestrator of innate infiltration, likely through its documented interactions with IRF3 and IRF7, which reinforce the downstream transcriptional program of the STING axis (Chen et al., 2020).

### 4.2 Mediating the proteasomal degradation of the cGAS sensor

Conversely, malignancies have evolved divergent TRIM-centered strategies to dismantle cGAS–STING signaling, ensuring that “danger signals” remain immunologically silent. The most direct strategy involves the ubiquitin-mediated clearance of the cGAS sensor itself. In microsatellite stable (MSS) gastric cancer—a phenotype notoriously resistant to immunotherapy—TRIM6 catalyzes the K27-linked polyubiquitination of cGAS, leading to its rapid proteasomal degradation. This non-canonical ubiquitination effectively abrogates type I interferon (IFN-I) secretion and prevents the recruitment of CD8^+^ T cells (Niu et al., 2025). A more complex mode of “metabolic hijacking” is observed in hepatocellular carcinoma (HCC), where the metabolic enzyme IDI1 functions as a non-metabolic adaptor. IDI1 recruits the E3 ligase TRIM41 to the cGAS complex, facilitating its ubiquitination and subsequent degradation. This IDI1–TRIM41–cGAS axis exemplifies how tumor cells repurpose metabolic components to neutralize innate sensors, ultimately reducing the expression of critical chemokines such as CCL5 and CXCL10 (Fu et al., 2024).

### 4.3 Indirect inhibition and epigenetic silencing of cGAS-STING signaling

TRIM-mediated inhibition also extends to the indirect modulation of upstream rheostats and downstream chromatin accessibility. TRIM27 attenuates the cGAS–STING axis by targeting the AMPK α1 subunit for K48-linked degradation (Wang L. et al., 2025). Since AMPK-mediated phosphorylation is essential for maintaining cGAS enzymatic activity, its depletion via TRIM27 serves as a metabolic “off-switch” for DNA sensing in ovarian cancer. Downstream of the sensor, TRIM proteins regulate the stability of the signaling adaptors and the transcription of effector genes. TRIM21 expression in HCC correlates negatively with global cGAS–STING activity, suggesting its role as a persistent brake on the pathway (Qi et al., 2020). In myeloid cells, TRIM33 acts as a chromatin reader that directly binds to the Ifnb1 gene promoter, imposing a transcriptional block that limits the efficacy of radiotherapy (Assouvie et al., 2025). Finally, TRIM28 modulates the axis by catalyzing the K63-linked ubiquitination of TBK1 (Ma et al., 2023). Interestingly, in the context of gastric cancer, this modification diverts the signal toward TBK1–mTOR signaling rather than the canonical IFN-I path, favoring PD-L1 upregulation and immune evasion over activation.

The deployment of multiple TRIM family members to target the cGAS–STING axis reflects a strategy of strategic encirclement. By intervening at the levels of metabolic priming (TRIM27), protein stability (TRIM6, TRIM41), spatial scaffolding (TRIM10), and chromatin accessibility (TRIM33), TRIM proteins ensure a comprehensive and redundant silencing of innate sensing. This multi-nodal regulation explains why single-target therapies often fail to “inflame” cold tumors, as the inhibition of one TRIM member may be compensated by another within the same axis. Consequently, targeting these E3 ligases—particularly those involved in K27-linked degradation or non-metabolic recruitment—offers a promising pharmacological frontier to restore the endogenous adjuvanticity of the tumor microenvironment.

## 5 The ubiquitin code in TRIM-Mediated immunomodulation

The functional versatility of TRIM proteins in tumor immunity is fundamentally dictated by the specific topology of the ubiquitin chains they assemble on target substrates (Table 1). K48-linked polyubiquitination serves as the canonical signal for proteasomal degradation, acting as a molecular rheostat to modulate immune response intensity. This degradative pathway bolsters anti-tumor immunity by eliminating immunosuppressive factors, such as the TRIM8-mediated clearance of MYOF (Wei et al., 2025) and the TRIM21-dependent degradation of METTL3 (Sun et al., 2023). Conversely, tumors co-opt this mechanism to facilitate immune evasion through the targeted depletion of essential immune activators. For instance, TRIM29 degrades the signaling adaptor TAB2 to suppress NK cell cytotoxicity (Dou et al., 2019), while TRIM27 targets the AMPK α1 subunit to attenuate cGAS-STING signaling (Wang L. et al., 2025).

**TABLE 1:** Systematic overview of the context-dependent, dual regulatory functions of TRIM family proteins in tumor immunity. This table delineates the specific target substrates, the exact type of ubiquitin linkage or modification (e.g., degradative K48-linked, non-proteolytic K63-linked, K27-linked polyubiquitination, or SUMOylation), and the resulting immunological outcomes across diverse tumor microenvironments and cell lineages.

TRIM protein	Substrate/Target	Modification/Linkage type	Functional outcome in tumor immunity	Tumor context/Cell type	References
TRIM2	IκBα	Proteasomal degradation	Induces sustained NF-κB activation and drives M2 macrophage polarization	Colorectal cancer/TAMs	Liu et al. (2024c)
TRIM3	TLR3	K63-linked polyubiquitination	Stabilizes TLR3 to sustain IFN-β production and reprogram the TME	NSCLC/TME	Xu et al. (2026)
TRIM3	Importin α3, α-ACTN4	Proteasomal degradation	Suppresses NF-κB signaling and restricts lymphangiogenesis	Gastric cancer, ESCC	Zhu et al. (2019)
TRIM5	IRF3/IRF7	Complex formation	Restricts monocyte infiltration and remodels the innate immune landscape	Glioma	Chen et al. (2020)
TRIM6	cGAS	K27-linked polyubiquitination	Mediates cGAS degradation, abrogating IFN-I secretion and CD8^+^ T cell recruitment	MSS Gastric cancer	Niu et al. (2025)
TRIM8	MYOF	K48-linked polyubiquitination	Targets Myoferlin for degradation to inhibit MMP secretion and metastasis	Lung cancer/M1 Macrophages	Wei et al. (2025)
TRIM10	STING	Spatial scaffolding	Facilitates STING aggregation in the Golgi for enhanced IFN-I production	Macrophages	Kong et al. (2023)
TRIM11	JAK1	K63-linked polyubiquitination	Inhibits JAK1 kinase activity, blunting IFN-γ-induced PD-L1 expression	Pan-cancer/Tumor cells	Xu et al. (2025)
TRIM14	PD-L1	Deubiquitination (Removal of K63-linked chains)	Recruits USP14 to prevent p62-mediated autophagic degradation of PD-L1	Breast cancer/Tumor cells	Liu et al. (2024b)
TRIM16	Galectin-3 (Gal-3)	Proteasomal degradation	Clears extracellular Gal-3 to protect CD8^+^ T cells from cuproptosis	Glioma/TME	Wang et al. (2024)
TRIM21	PD-L1	Proteasomal degradation	Directly promotes PD-L1 turnover to enhance immunosurveillance	NSCLC, LUAD/Tumor cells	Gao et al. (2021), Sun et al. (2023)
TRIM21	STAT1	Proteasomal degradation	Prevents STAT1 accumulation, maintaining TAM homeostasis	Gastric cancer/TAMs	Zhang et al. (2025)
TRIM21	METTL3	K48-linked polyubiquitination	Induces ferroptotic stress, leading to compensatory PD-L1 upregulation	PDAC/Tumor cells	Mao et al. (2025)
TRIM21	PD-1	K63-linked polyubiquitination	Stabilizes surface PD-1 to sustain T cell exhaustion	T cells	Shi et al. (2025)
TRIM22	Cytokine receptors/Signaling molecules	Positive regulation (Mechanism elusive)	Positively correlates with the infiltration of lymphocytes and dendritic cells	Colon adenocarcinoma (COAD)	Guo et al. (2021)
TRIM24	SMAD3	Transcriptional complex	Enhances CXCL2 expression and recruits MDSCs	TNBC/MDSCs	Yu et al. (2021)
TRIM25	VISTA	K63-linked polyubiquitination	Stabilizes the VISTA checkpoint to establish an immunosuppressive barrier	T cells	Sun et al. (2025)
TRIM25	Unknown (NF-κB axis)	Signaling activation	Inhibits apoptosis and concurrently upregulates PD-L1	Glioma/Macrophages	Ge et al. (2022)
TRIM27	AMPK α1	K48-linked polyubiquitination	Induces AMPK degradation, attenuating cGAS–STING activity	Ovarian cancer/Tumor cells	Wang et al. (2025b)
TRIM28	RIPK1	K63-linked polyubiquitination	Activates NF-κB-dependent CXCL1 expression to recruit MDSCs	NSCLC/MDSCs	Liang et al. (2023)
TRIM28	PD-L1	SUMOylation/Suppresses ubiquitination	Stabilizes PD-L1 via B-box2 domain physical interaction	Gastric cancer/Tumor cells	Ma et al. (2023)
TRIM28	TBK1	K63-linked polyubiquitination	Diverts signaling toward TBK1–mTOR pathways for PD-L1 transcription	Gastric cancer/Tumor cells	Ma et al. (2023)
TRIM28	PARP1	SUMOylation	Hyperactivates PARP1 to deplete nuclear NAD^+^ and compromise CD8^+^ T cell function	ccRCC/TME	Zhan et al. (2025)
TRIM29	IGF2BP1	Proteasomal degradation	Destabilizes PD-L1 mRNA to relieve T cell suppression	Gastric cancer/Tumor cells	Jiang et al. (2024b)
TRIM29	TAB2	K48-linked polyubiquitination	Degrades TAB2 to dampen NF-κB/MAPK signaling and NK cell cytotoxicity	Melanoma/NK cells	Dou et al. (2019)
TRIM32	XIAP	Proteasomal degradation	Sensitizes tumors to TNFα-induced apoptosis	Pan-cancer/Tumor cells	Ryu et al. (2011)
TRIM32	PDE9A	Protein interaction	Recruits and polarizes M2-like TAMs *via* PI3K/AKT signaling	Gastric cancer/TAMs	Wang et al. (2025a)
TRIM33	Ifnb1 promoter	Chromatin reading/Repression	Directly represses IFN-β transcription to limit radiotherapy efficacy	Myeloid cells	Assouvie et al. (2025)
TRIM35	LSD1	K63-linked polyubiquitination	Inhibits LSD1 activity to stabilize IFNGR1 and recruit CD8^+^ T cells	NSCLC/Tumor cells	Tang et al. (2023)
TRIM35	CLOCK	Proteasomal degradation	Alleviates CLOCK-mediated suppression to enhance NK cell infiltration	DLBCL/NK cells	Tan et al. (2021)
TRIM41	cGAS	Proteasomal degradation	Abrogates DNA sensing and reduces chemokine production (CCL5/CXCL10)	Hepatocellular carcinoma	Fu et al. (2024)
TRIM59	PIAS1-STAT1	Complex formation	Inhibits STAT1 to abrogate anti-tumor effector production	Macrophages (Intra.)	Su et al. (2020)
TRIM59	ABHD5	Proteasomal degradation	Triggers NLRP3 inflammasome activation and IL-1β release	Macrophages (Exo.)	Liang et al. (2020)
TRIM59	PI3K/ERK signaling components	Enzymatic suppression	Acts as a metastatic surveillance factor in M2 macrophages to inhibit migration and invasion	Melanoma/M2 Macrophages	Tian et al. (2019a)
TRIM59	Tumor cell membrane	Direct physical contact	Upregulated upon BCG stimulation to induce direct pro-apoptotic effects on tumor cells	Fibrosarcoma/Activated Macrophages	Tian et al. (2019b)
TRIM65	JAK1/STAT1 axis	Unknown inhibition	Prevents immunostimulatory M1 polarization	Hepatocellular carcinoma/TAMs	Jiang et al. (2024a)

In contrast, K63-linked ubiquitination exerts non-proteolytic effects, primarily governing protein stabilization, enzymatic inhibition, and the assembly of signaling scaffolds. This linkage type is critical for maintaining the structural integrity of immune sensors and checkpoints. TRIM3-mediated K63 ubiquitination stabilizes TLR3 to sustain IFN-β production (Xu et al., 2026), whereas TRIM25 and TRIM21 utilize K63 chains to stabilize the immune checkpoints VISTA (Sun et al., 2025) and PD-1 (Shi et al., 2025) on T cells, respectively, driving immunosuppression. K63 linkages also directly modulate enzymatic activity without inducing degradation. TRIM11 catalyzes the K63-linked ubiquitination of JAK1 to inhibit its kinase function, dismantling the IFN-γ/PD-L1 axis (Xu et al., 2025), while TRIM35 inhibits the histone demethylase LSD1 to epigenetically reprogram the tumor microenvironment (Tang et al., 2023).

Expanding beyond classical linkages, TRIM proteins leverage non-canonical ubiquitin chains and ubiquitin-like modifiers to subvert immune surveillance. K27-linked ubiquitination plays a specialized role in innate immune evasion, notably through the TRIM6-dependent degradation of the cytosolic DNA sensor cGAS (Niu et al., 2025). The TRIM network also intersects intimately with SUMOylation and deubiquitination pathways to fine-tune checkpoint stability. TRIM28 facilitates the SUMOylation of both PD-L1 and PARP1 to enforce an immunosuppressive niche (Zhan et al., 2025), whereas TRIM14 recruits the deubiquitinase USP14 to strip K63-linked chains from PD-L1, preventing its autophagic clearance (Liu D. et al., 2024). By deploying this diverse structural code, TRIM proteins orchestrate a highly precise regulatory network that determines the ultimate trajectory of the anti-tumor immune response.

## 6 Conclusion and future prospects

The exploration of the Tripartite Motif (TRIM) family in cancer biology has traditionally centered on their direct regulation of tumor cell-intrinsic behaviors, such as proliferation, metabolic reprogramming, epithelial-mesenchymal transition (EMT), and metastasis (Zhou et al., 2014; Pathiraja et al., 2015; Zhang et al., 2018). However, as delineated in this review, the functional spectrum of TRIM proteins extends far beyond these classical oncogenic or tumor-suppressive roles. We propose that TRIM proteins should be reconceptualized as central architects of the tumor immune microenvironment (TME), capable of orchestrating the delicate balance between immune surveillance and immune evasion. Our synthesis of recent findings reveals that TRIM proteins modulate tumor immunity through three primary dimensions: the direct alteration of immune cell phenotypes (e.g., macrophage polarization and T/NK cell cytotoxicity), the regulation of immune checkpoints (PD-L1/PD-1/VISTA), and the multi-nodal remodeling of innate sensing pathways, most notably the cGAS-STING axis. Crucially, this regulatory versatility is fundamentally dictated by a sophisticated “ubiquitin code.” The specific topology of ubiquitin linkages—ranging from degradative K48-linked and K27-linked chains to non-proteolytic K63-linked scaffolds—serves as the molecular logic that determines whether a TRIM protein acts as an immune sensitizer or a suppressor. By integrating these diverse structural signals, the TRIM family stands as a decisive regulatory hub upstream of critical immunotherapy targets (Dou et al., 2019; Qi et al., 2020; Gao et al., 2021; Sun et al., 2023; Jiang T. et al., 2024; Mao et al., 2025; Shi et al., 2025).

Although the macroscopic classification of the TRIM family delineates broad immunostimulatory or immunosuppressive roles, the fundamental complexity of ubiquitin-mediated immune regulation is best illustrated by a select group of highly plastic hub proteins. The dual functions of these pivotal molecules are stringently governed by three specific dimensions: cell-lineage specificity, substrate preference, and the signaling microenvironment. TRIM21 exemplifies this context-dependent duality. It sensitizes tumor cells to immunosurveillance via direct PD-L1 degradation (Gao et al., 2021; Sun et al., 2023) but paradoxically enforces immune exhaustion within the lymphoid compartment by stabilizing surface PD-1 through K63-linked ubiquitination (Shi et al., 2025). A parallel divergence occurs with TRIM29, which functions as a tumor suppressor in gastric cancer by destabilizing PD-L1 transcripts and simultaneously acts as a negative checkpoint in natural killer cells by degrading TAB2 to restrict cytotoxicity (Dou et al., 2019; Jiang T. et al., 2024). TRIM32 exhibits similar functional polarity, facilitating immune-mediated clearance by ubiquitinating the anti-apoptotic protein XIAP (Ryu et al., 2011) in malignant cells while driving M2 macrophage polarization through the stromal PDE9A/PI3K/AKT axis (Wang C. et al., 2025). Furthermore, key regulators such as TRIM25 and TRIM59 orchestrate multi-compartmental immune evasion networks. TRIM25 coordinates tumor-intrinsic PD-L1 upregulation with T cell-intrinsic VISTA stabilization (Sun et al., 2025), whereas TRIM59 demonstrates extensive spatial versatility, mediating processes from intracellular STAT1 inhibition to exosome-driven inflammasome activation (Su et al., 2020). The net immunological outcome within the tumor microenvironment ultimately relies on the dynamic integration of these opposing signals. Prioritizing the mechanistic duality of these core regulators transitions the current paradigm from a descriptive overview to a mechanism-driven synthesis. This perspective establishes an essential theoretical foundation for developing context-aware targeted interventions, including precise proteolysis-targeting chimeras (PROTACs).

Targeting the pivotal regulation of the PD-1/PD-L1 axis and cGAS–STING pathway by TRIM proteins offers a compelling pharmacological frontier to overcome immunotherapy resistance and inflame “cold” tumors. Therapeutic strategies potentiating PD-L1-degrading members, such as TRIM21 and TRIM3, or inhibiting stabilizers like TRIM14 and TRIM28 could synergize with checkpoint blockade, while their intrinsic E3 ligase activity positions them as ideal effectors for proteolysis-targeting chimeras (PROTACs). However, clinical translation is currently impeded by the high structural conservation among more than 80 family members, which complicates selective drug design, and a reliance on bulk analysis that obscures cell-type-specific functions. To overcome these limitations, future investigations must pivot toward comprehensive multi-omics integration strategies. Recent seminal studies demonstrate the substantial translational potential of integrating single-cell RNA sequencing (scRNA-seq) with bulk RNA-seq. For instance, a multi-omics investigation in melanoma integrated single-cell and bulk transcriptomic data to delineate the prognostic value of B cell and macrophage infiltration, successfully constructing a machine learning model to predict immune checkpoint inhibitor (ICI) efficacy (Zhang Y. et al., 2024). Similarly, combining scRNA-seq with global immune subtyping in hepatocellular carcinoma has effectively revealed cellular heterogeneity alongside specific cell types that may dictate ICI responsiveness (Lai et al., 2025). Systematically applying these multi-omics frameworks to TRIM family research will enable the precise resolution of their immunoregulatory functions within specific microenvironmental and cell-lineage contexts.

Notably, most current findings rely on observational studies that associate TRIM expression profiles and immune cell infiltration with patient survival. To effectively translate these multifaceted interactions into actionable clinical targets, the field must move beyond mere correlations and rigorously establish definitive causal relationships between TRIM family genes and disease prognosis. Future research should prioritize sophisticated causal inference strategies to address this gap. Mendelian randomization (MR) leverages genetic variants as instrumental variables to provide a robust analytical framework for evaluating causal links between specific biological exposures and clinical outcomes, minimizing confounding biases (Li et al., 2024). Furthermore, cutting-edge integrative approaches, such as combining large-scale two-sample MR with single-cell transcriptomics, have recently proven highly effective in mapping causal networks down to specific functional cell subpopulations (Zhang C. et al., 2024). By employing these advanced analytical frameworks, future studies can systematically determine whether specific TRIM proteins are genuine drivers of immune evasion or merely prognostic markers. This paradigm shift from correlation to causation is essential for identifying the most viable TRIM candidates for targeted immunotherapy.

Translating these molecular discoveries into actionable clinical tools ultimately depends on developing robust biomarkers. As precision oncology advances, there is an urgent need to develop TRIM-based biomarkers capable of accurately predicting patient prognosis and immunotherapeutic responses. In this context, modeling approaches based on immune-related gene pairs (IRGPs) offer a highly promising strategy for constructing robust prognostic signatures (Xie et al., 2022). This intra-sample evaluation effectively eliminates batch effects across multicenter cohorts and technical biases introduced by disparate sequencing platforms. Future research must prioritize screening core TRIM family members and integrating them with other immune-related genes to construct specific IRGP prognostic models. These TRIM-based paired signatures hold the potential to accurately stratify patients into high-risk and low-risk relapse subpopulations across diverse clinical cohorts while effectively predicting immune cell infiltration within the TME. Developing such predictive biomarkers will enable clinicians to more accurately identify specific patient populations poised to benefit from immune checkpoint blockade (ICB) therapy. Ultimately, this approach translates the deep mechanistic understanding of the dual roles of TRIM proteins into next-generation, personalized combinatorial immunotherapies.
